# Resolving tooth development at single-cell resolution: advancing dental regeneration

**DOI:** 10.3389/fcell.2026.1785805

**Published:** 2026-03-12

**Authors:** Chengcheng Liao, Jun Cai, Yanmei Liu, Linlin Xiao, Mingli Xiang, Man Qu, Qian Long, Meiling Xiang, Sicen Long, Houli Peng, Jianguo Liu, Xiaoyan Guan

**Affiliations:** 1 Department of Orthodontics II, Affiliated Stomatological Hospital of Zunyi Medical University, Zunyi, China; 2 Key Laboratory of Oral Disease Research (Guizhou Provincial Department of Education & Zunyi City), School of Stomatology, Zunyi Medical University, Zunyi, China; 3 West China School of Stomatology, Sichuan University, Chengdu, China

**Keywords:** dentin, enamel, regenerative medicine, single-cell sequencing, tooth development

## Abstract

Tooth development is a continuous, highly orchestrated process that serves as an ideal model for dissecting the cellular and molecular mechanisms of organogenesis. In recent years, advances in single-cell transcriptomics have provided unprecedented insights into cellular heterogeneity, lineage trajectories, and molecular signaling networks during mouse and human tooth development, greatly enhancing our understanding of odontogenesis. Crucially, these single-cell-level studies of tooth development provide both a theoretical foundation and advanced strategies for dental tissue regeneration.

## 1 Introduction

The formation of teeth represents a paradigm of organogenesis, in which complex epithelial-neural crest mesenchymal interactions and tightly coordinated developmental signaling events give rise to the highly organized structures of enamel, dentin, cementum, pulp, and periodontal ligament (Bei, 2009). However, dental caries (Selwitz et al., 2007), periodontitis (Michaud et al., 2017), pulp and periapical inflammation (Galler et al., 2021), as well as traumatic injuries (Tsai et al., 2017) frequently result in the loss of dental and periodontal tissues, and severe cases can lead to tooth loss, thereby compromising essential functions such as mastication, speech, airway maintenance, and the structural support of facial soft tissues (Wang et al., 2024). Currently, therapeutic approaches such as root canal treatment (RCT), periodontal scaling, guided tissue regeneration (GTR), and dental implantation represent the main clinical strategies for managing these oral diseases (Del Fabbro et al., 2016; Liu et al., 2022). Despite these advanced therapeutic approaches, the complete restoration of dental, pulpal, and periodontal function remains a major challenge (Zeichner-David, 2006; Balic, 2018). Stem cell-based, biomimetic material-driven, and bioactive factor-mediated tissue engineering strategies offer promising avenues for the functional regeneration of dental, pulpal, and periodontal tissues, and potentially even the whole tooth (Zhai et al., 2019).

Over the past decades, our understanding of tooth development has advanced substantially, with the roles of multiple key cell populations elucidated through mouse genetic models. Studies on these populations and their fate-determining mechanisms have provided new insights and potential strategies for tooth regeneration. For example, a Runx2^+^/Gli1^+^ subpopulation identified in adult mouse incisors regulates the proliferation and differentiation of transit-amplifying cells (TACs), as well as the rate of incisor growth, via insulin-like growth factor (IGF) signaling (Chen H et al., 2020), thereby providing insights into the maintenance and regenerative capacity of the local microenvironment during tooth development. He et al. (2021) identified an Lhx6^+^ dental mesenchymal subpopulation that regulates root furcation formation by modulating Wnt signaling activity within the local microenvironment, providing important insights into the morphogenesis of dental roots and informing the development of root regeneration strategies. Despite sustained efforts to dissect the cellular and molecular basis of tooth formation (Ahtiainen et al., 2016), the extent of cellular heterogeneity and the dynamic changes in key developmental signals during odontogenesis remain incompletely explored.

Single-cell RNA sequencing (scRNA-seq) enables the profiling of genetic information at the individual cell level, offering significant advantages in identifying complex and rare cell populations, investigating intercellular interactions, and tracing the developmental trajectories of distinct cell types (Hwang et al., 2018). At present, scRNA-seq has found extensive applications in dental and oral research, encompassing tooth, pulp, and periodontal biology (Wu et al., 2022). Particularly in the field of tooth development, scRNA-seq has been used to construct single-cell atlases of developing dental tissues in both humans and mice (Jiang et al., 2025). Collectively, these investigations not only furnish deeper and more nuanced insights into the cellular and molecular mechanisms underlying tooth development but also propel advances in dental regenerative strategies.

Against this background, in the present review, we summarize how heterogeneous cellular populations within dental tissues contribute to tooth development and discuss the potential implications of these findings for regenerative dentistry.

## 2 Tissue architecture underlying tooth development

Similar to most ectoderm-derived organs, tooth morphogenesis is driven by continuous, finely tuned epithelial–mesenchymal interactions. The process is initiated by the thickening of the oral epithelium, which subsequently forms the dental lamina endowed with odontogenic potential. Remarkably, at this stage, recombining the dental lamina epithelium with non-dental mesenchyme is sufficient to autonomously initiate tooth formation (Lumsden, 1988; Mina and Kollar, 1987). Subsequently, the dental lamina epithelium invaginates into the underlying cranial neural crest (CNC)-derived mesenchyme to form the tooth bud, with dental mesenchymal cells condensing around the epithelial tooth germ. The epithelium then transfers its odontogenic potential to the mesenchymal compartment, orchestrating the subsequent stages of tooth development (Kollar and Baird, 1970; Thesleff and Sharpe, 1997). The epithelial component differentiates into enamel under the influence of various signaling factors, with its folding patterns determining both the shape and number of cusps (Jernvall and Thesleff, 2000). The mesenchymal compartment further differentiates into the dental papilla and dental follicle. Situated beneath the enamel, the dental papilla gives rise to dentin and contributes to root development and elongation (Yao et al., 2024). As root formation progresses, the portion of the dental papilla enclosed by the enamel and dentin of the crown continues to mature into the dental pulp, a highly vascularized and innervated structure (Yao et al., 2024). The dental follicle, enveloping the developing enamel, dentin, and dental papilla, is responsible for forming cementum, the lamina dura (alveolar bone), and the periodontal ligament, and serves as a critical effector tissue for tooth eruption (Zhou et al., 2019).

Overall, tooth morphogenesis exemplifies how dynamic epithelial-mesenchymal crosstalk orchestrates complex tissue patterning, offering a conceptual framework for understanding organogenesis and guiding regenerative strategies.

## 3 Morphogenesis and regenerative of enamel

Enamel, located coronally to the dentin, is the hardest tissue in the human body. In certain animals, such as mouse incisors, enamel epithelial stem cells continuously give rise to functional ameloblasts, enabling the regeneration of enamel (Binder et al., 2020). This feature renders mouse incisors an ideal model for studying tooth development. However, substantial differences remain between mouse and human dentition, including variations in cusp shape and number, as well as differences in the timing and sequence of molar development (Kavanagh et al., 2007). In humans, the enamel lacks a stem cell niche, rendering it incapable of self-repair once damaged (Fugolin and Pfeifer, 2017).

Beyond the ameloblast lineage, enamel-forming organs also comprise multiple supporting cell populations, including stellate reticulum cells and inner and outer enamel epithelial cells (Wu et al., 2024). These supporting cells are considered essential for maintaining ameloblast function (Harada et al., 2006; Maas and Bei, 1997). However, prior to the advent of scRNA-seq, the roles of these supporting cells in ameloblast differentiation and functional maturation remained largely unclear. Recent scRNA-seq studies (Alghadeer et al., 2023; Krivanek et al., 2020; Sharir et al., 2019) have begun to elucidate the molecular signatures and differentiation trajectories of heterogeneous epithelial populations during human and mouse tooth germ development, and have proposed novel strategies for human enamel regeneration (Alghadeer et al., 2023).

### 3.1 Cellular architecture underlying human enamel formation

Alghadeer et al. (2023) employed scRNA-seq to investigate human fetal tooth development between 9 and 22 gestational weeks, identifying 13 epithelial cell populations involved in enamel formation: oral epithelium (OE), dental epithelium (DE), enamel knot (EK), outer enamel epithelium (OEE), inner enamel epithelium (IEE), cervical loop (CL), inner stratum intermedium (SII), outer stratum intermedium (SIO), inner stellate reticulum (SRI), outer stellate reticulum (SRO), pre-ameloblasts (PA), early ameloblasts (eAM), and secretory ameloblasts (sAM).

**FIGURE 1:**
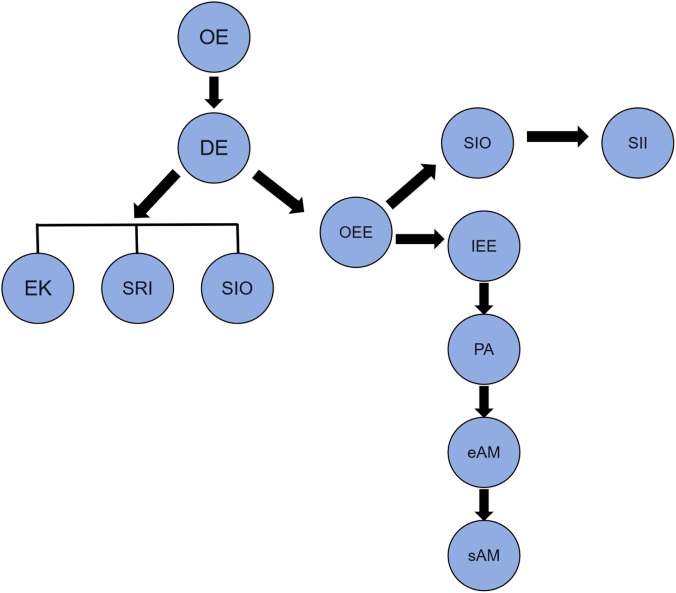
The OE directly differentiates into DE, which subsequently gives rise to the EK, SIO and SRI; meanwhile, the OEE lineage generates SII, SIO, IEE, PA, eAM, and sAM cells.

Pseudotime analysis indicated that the OE directly differentiates into DE, which subsequently gives rise to the EK and stellate reticulum lineages (SIO and SRI); meanwhile, the OEE lineage generates SII, SIO, IEE, PA, eAM, and sAM cells (Alghadeer et al., 2023) (Figure 1). In another scRNA-seq study of human tooth germs at 17–24 gestational weeks, it was found that OEE differentiates into IEE, which subsequently gives rise to an activated leukocyte cell adhesion molecule (ALCAM)^+^ stem cell population with the capacity to generate stratum intermedium cells (Shi et al., 2024). In addition, the EK has been identified as an indispensable signaling center during human tooth formation, playing a pivotal role in crown morphogenesis (Alghadeer et al., 2023). Supporting cells, such as SII (via Hedgehog and Wnt pathways) and SIO (via the TGF-β pathway), appear to exert precise signaling influences on specific neighboring epithelial cell populations within the ameloblast lineage (Alghadeer et al., 2023).

Alghadeer et al. (2023) identified Leucine Rich Repeat Containing G Protein-Coupled Receptor 6 (LGR6)^+^ cells localized at the CL at the junction of the OEE and IEE. Previous studies have reported that the CL region in adult mouse incisors harbors epithelial stem cells (Chang et al., 2013). LGR6^+^ cells at the CL can give rise to the Hertwig’s epithelial root sheath (HERS), playing a critical role in root formation, and may also contribute to ameloblast formation during the early stages of crown development (Alghadeer et al., 2023). (summarized in Table 1). However, human enamel development lacks a continuously maintained CL niche, representing a fundamental limitation for *in vivo* enamel regeneration. This underscores both the conservation of epithelial signaling hierarchies across species and the species-specific differences that must be carefully considered when translating regenerative strategies from mouse models to humans.

**TABLE 1:** Epithelial cell subpopulations in human tooth development and their lineage relationships.

Cell type	Markers	Function	Lineage relationship	References
Oral epithelium	Not mentioned	The outermost layer of initiating epithelial cells	Directly differentiates into DE.	Alghadeer et al. (2023)
Dental epithelium	Not mentioned	Early dental epithelium, the starting point for further specialization	Gives rise to the EK and stellate reticulum lineage cells (SIO, SRI)	Alghadeer et al. (2023)
Enamel knot	SHH^+^, WNT10A^+^, WNT10B^+^	A signaling center during development, determining tooth crown shape	Secretes signaling molecules such as SHH, WNT10 A/B, regulating surrounding cells	Alghadeer et al. (2023), Shi et al. (2024)
Outer Enamel epithelium	Not mentioned	Epithelial layer located on the outer convex aspect of the tooth germ	Gives rise to subsequent differentiating cells including SII, SIO, and IEE.	Alghadeer et al. (2023)
Inner Enamel epithelium	SP6^+^ (cytoplasmic), ALCAM^+^ (subpopulation)	Epithelial cells located on the inner concave aspect, adjacent to the dental papilla	Differentiates into PA and contains ALCAM^+^ stem cell populations	Alghadeer et al. (2023), Shi et al. (2024)
Cervical loop	LGR6^+^	The looped region at the junction of the OEE and IEE, containing stem cells	LGR6^+^ cells localize here; can form HERS, involved in root formation	Alghadeer et al. (2023), Chang et al. (2013)
Inner stellate reticulum	TGF-β ligand^+^	Support cells located on the inner side of the stellate reticulum	Supports adjacent epithelial cells via the TGF-β pathway	Alghadeer et al. (2023)
Outer stellate reticulum	Not mentioned	Support cells located on the outer side of the stellate reticulum	Supports adjacent epithelial cells via the TGF-β pathway	Alghadeer et al. (2023)
Inner stratum intermedium	HH ligand^+^, EGF^+^ (late stage)	Inner stratum intermedium cells adjacent to IEE/PA.	Supports the ameloblast lineage via HH and WNT pathways; secretes EGF at later stages	Alghadeer et al. (2023)
Outer stratum intermedium	TGF-β ligand^+^, FGF^+^ (late stage)	Outer stratum intermedium cells adjacent to OEE.	Supports the ameloblast lineage via the TGF-β pathway; secretes FGF at later stages	Alghadeer et al. (2023)
Pre-ameloblasts	HH ligand^+^, SP6^+^ (cytoplasmic)	IEE-derived precursors poised to become mature ameloblasts	Secrete HH ligands; transition into eAM.	Alghadeer et al. (2023)
Early ameloblasts	WNT ligand^+^, SP6^+^ (nuclear translocation)	Early stage of ameloblast differentiation	Transition into sAM); secrete WNT ligands	Alghadeer et al. (2023)
Secretory ameloblasts	AMBN^+^, AMELX^+^, SP6^+^ (nuclear)	Functionally mature ameloblasts that secrete enamel matrix	Final differentiation stage, responsible for enamel formation	Alghadeer et al. (2023)

### 3.2 Single-cell insights into molecular signaling during human enamel morphogenesis

Using scRNA-seq, Alghadeer et al. (2023) found that during the transition from OE to DE, underlying dental mesenchymal cells secrete BMP, ACTIVIN, and non-canonical WNT ligands, whereas canonical WNT ligands are produced within the OE. Similarly, during the transition from DE to OEE, DE and the EK secrete WNT ligands, whereas BMP and FGF ligands are primarily derived from the dental mesenchyme (Alghadeer et al., 2023). Moreover, Shi et al. (2024) reported that the EK secretes SHH, WNT10A, and WNT10B. These findings underscore the pivotal role of the EK as a signaling hub during the early stages of enamel development.

During the transition from OEE to IEE, the underlying dental papilla (mesenchyme) primarily influences ameloblast differentiation through the secretion of BMPs (Alghadeer et al., 2023). Notably, interactions between pre-odontoblasts (POB), odontoblasts (OB), and epithelial cells are particularly prominent, with the secretion of FGF and BMP promoting the transition of PA into eAM or sAM (Alghadeer et al., 2023). During the late stages of ameloblast differentiation, PA) and SII secrete Hedgehog (HH) ligands, while SRI and SIO produce TGF-β ligands. Subsequently, in the final maturation from eAM to secretory ameloblasts (sAM), WNT ligands are predominantly secreted by eAM cells, EGF by SII, and FGF by SIO (Alghadeer et al., 2023). The secretion of these ligands not only promotes ameloblast maturation but may also influence mesenchymal tissue development. Overall, WNT, TGF-β, HH, FGF, and BMP signaling pathways are the most active during ameloblast differentiation, highlighting the critical role of mesenchymal cells in orchestrating epithelial tissue development. While scRNA-seq has revealed potential signaling roles of SII, SIO, and SRI cells in ameloblast differentiation (Alghadeer et al., 2023), functional validation *in vivo* remains sparse. Future work should aim to manipulate these populations to confirm their regulatory contributions.

During the transition from outer enamel epithelium (OEE) to inner enamel epithelium (IEE), WNT activity correlates with SP6 expression (Alghadeer et al., 2023). SP6 is initially localized in the cytoplasm of IEE and PA cells, subsequently expressed in eAM and sAM, and eventually translocates to the nucleus where it co-localizes with AMBN expression (Alghadeer et al., 2023). A previous study reported that WNT signaling can induce the expression of the transcription factor SP6 (Aurrekoetxea et al., 2016), which in turn acts on the promoters of AMBN and AMELX (Rhodes et al., 2021). This finding underscores the critical role of WNT-induced SP6 expression in orchestrating ameloblast maturation.

Across human enamel development, multiple signaling pathways coordinate ameloblast differentiation. Early OE-to-DE transitions are regulated primarily by mesenchyme-derived BMP/ACTIVIN and OE-derived canonical WNT ligands, whereas EK-derived SHH and WNT10 A/B signals orchestrate crown morphogenesis. Notably, SII and SIO supporting cells modulate TGF-β and HH pathways in a spatially restricted manner. Integrating these data, we propose a hierarchical model in which mesenchymal signals initiate lineage specification, epithelial cross-talk refines differentiation, and supporting cell niches fine-tune maturation. This framework highlights potential targets for enamel regeneration while underscoring the challenge of replicating human-specific signaling environments *in vitro*.

### 3.3 Cellular foundations underlying mouse incisor enamel formation

**FIGURE 2:**
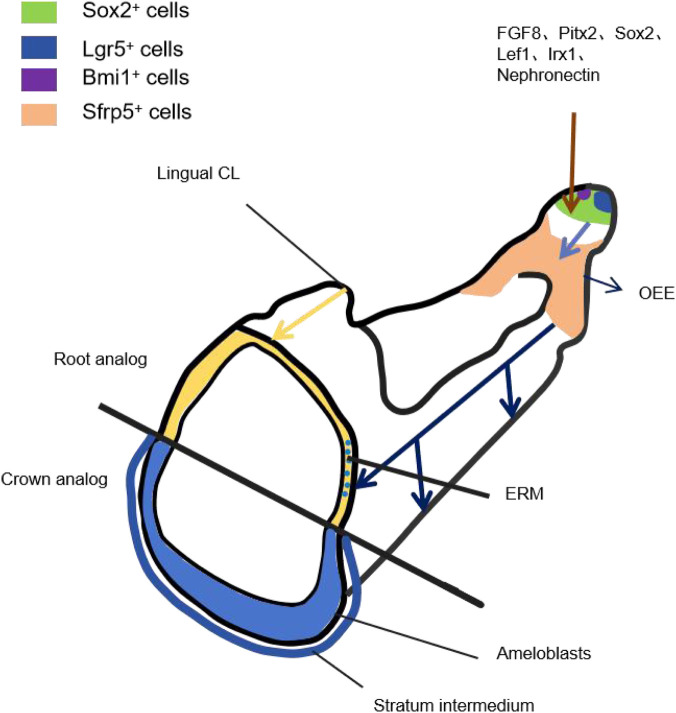
During the development of mouse incisors, Sox2^+^ stem cells regulated by FGF8 signaling are specifically located in the dental follicle. Sox2^+^ is modulated by regulatory factors such as Pitx2, Sox2, Lef1, Irx1, Nephronectin and Bmi1^+^, and Lgr5^+^ cells are identified as a subpopulation within the Sox2^+^ region. They are the first cells to re-proliferate after the loss of Sox2^+^ cells. Sox2^+^ stem cells generate Sfrp5^+^ progenitor cells, which eventually differentiate into ameloblasts and SI, SR and OEE cells, and participate in the renewal process of the epithelial remnant tissue (ERM). It generates all the epithelial lineages involved in enamel formation, thereby promoting the continuous enamel development of mouse incisors.

Histological analyses have traditionally identified four principal epithelial cell types in mouse incisors, including the IEE/OEE, SR, AM, and SI (Thesleff, 2003). Juuri et al. (2012) demonstrated that during mouse incisor development, Sox2^+^ stem cells, regulated by FGF8 signaling, are specifically localized to the CL. These cells give rise to Sfrp5^+^ progenitors, which in turn generate all epithelial lineages contributing to enamel formation, thereby driving continuous enamel development in mouse incisors. Sanz-Navarro et al. (2018) further explored the heterogeneity of Sox2^+^ stem cells and identified Lgr5^+^ cells as a subpopulation within the Sox2^+^ compartment, which are the first to repopulate following Sox2^+^ cell ablation. In addition, regulatory factors such as Pitx2, Sox2, Lef1, Irx1, and Nephronectin have been shown to be critically involved in maintaining the homeostatic balance of Sox2^+^ stem cells (Yu et al., 2020; Arai et al., 2017). Using lineage tracing, Biehs et al. (2013) identified Bmi1^+^ epithelial stem cells as key regulators of enamel development, acting through Bmi1-mediated repression of Ink4a/Arf and Hox gene expression to maintain proper enamel formation (Figure 2).

Krivanek et al. (2020) utilized Smart-seq2-based single-cell transcriptomic profiling to generate a comprehensive cellular atlas of the mouse incisor epithelium. Within the Krt14^+^/Cdh1^+^ epithelial compartment, they identified 13 transcriptionally distinct subpopulations. Among these, Shh^+^, Enam^+^, Klk4^+^, and Gm17660^+^ cells corresponded to the pre-secretory, secretory, maturation, and post-maturation stages of ameloblast differentiation, respectively—thereby reconstructing the full developmental trajectory of ameloblast lineage progression. Moreover, they demonstrated that SI cells play a pivotal role in maintaining the vascular-ameloblast interface, which is essential for proper enamel formation (Krivanek et al., 2020). Consistently, Chiba et al. (2020) through scRNA-seq analysis, further confirmed the presence of AM, IEE/OEE, and SI/SR populations within the mouse incisor epithelium, and refined the classification of sAM into Dspp^+^ and Ambn^+^ subclusters, representing early and fully differentiated sAM states, respectively.

Historically, it has been proposed that the slowly cycling epithelial stem cells located in the CL of mouse incisors give rise to IEE cells, which subsequently differentiate into all epithelial lineages contributing to enamel formation (Kuang-Hsien Hu et al., 2014). Sharir et al. (2019) used scRNA-seq to reveal that, during normal enamel development in mouse incisors, most IEE and SI cells differentiate into ameloblasts, while a small subset gives rise to SR and OEE cells. However, upon injury or disruption of homeostasis, SI cells acquire the capacity to directly convert into IEE and ameloblasts (Sharir et al., 2019). Mechanistically, NOTCH1 signaling serves as a key regulator mediating this conversion, whereas Cldn10 may facilitate ion permeability in SI cells, thereby promoting enamel formation (Chiba et al., 2020).

Taken together, these studies substantially advance our understanding of the cellular mechanisms sustaining continuous enamel formation in mouse incisors and may provide mechanistic insights into the etiology of human enamel developmental disorders. However, pronounced morphological and developmental disparities between mouse and human dentition constrain the direct extrapolation of these findings, and their validation or translational application in human enamel development and regenerative strategies remains largely uncharted.

### 3.4 Cellular foundations underlying enamel formation in mouse molars

Using scRNA-seq, Ye et al. (2022) identified four epithelial subpopulations in the mandibular epithelium of E12 mouse embryos: (1) Cxcl14^+^/Tfap2b^+^ aboral epithelium, extending from the anterior dental lamina to the ventral mandibular region; (2) Pitx2^+^/Irx2^+^ dental epithelium; (3) Rtl3^+^/Col14a1^+^ cells located posterior to the dental epithelium, corresponding spatially to the oral–lingual axis of the mandibular epithelium; and (4) Grhl3^+^/Irf6^+^ periderm cells. Notably, the Pitx2^+^/Irx2^+^ dental epithelium could be further subdivided into four subclusters: Timp3^+^/Prss23^+^ diastema, Ednrb^+^/Dtit4l^+^ incisors, Fgf8^+^ molars, and Gad1^+^/Sp5^+^/Proser2^+^ initiation knot (IK) (Ye et al., 2022).

The IK subpopulation exhibits key developmental signaling molecules, including Shh, Dkk4, and Fgf20, and is enriched for pathways that promote cell proliferation and tooth morphogenesis (Ye et al., 2022). Incisor and molar subpopulations display highly similar transcriptional profiles, expressing a range of dental epithelial markers such as Pitx2, Irx1, and Fst (Mucchielli et al., 1997; Yu et al., 2017), and can also be distinguished using newly identified markers, including Ntrk2, Dsc3, Enc1, and Osbpl6 (Ye et al., 2022). Diastema cells, located in the gap between the incisor and molar fields, likely undergo regression, contributing to the separation between adjacent teeth (Ye et al., 2022).

Collectively, these findings illuminate the cellular basis of early dental epithelium development in mice. Nevertheless, how these epithelial subpopulations subsequently contribute to molar enamel formation remains largely unexplored.

### 3.5 iPSC-derived ameloblast organoids and biomimetic enamel regeneration strategies

To model human enamel-related disorders *in vitro* and explore strategies for enamel regeneration, Alghadeer et al. (2023) established a serum-free, chemically defined differentiation protocol to derive human dental epithelial-like cells from induced pluripotent stem cells (iPSCs). Leveraging key developmental signaling cues identified through scRNA-seq, these iPSCs were further guided to form human ameloblast organoids. Upon co-implantation with human dental papilla stem cells beneath the renal capsule, the organoids generated mineralized structures expressing Ameloblastin, Amelogenin, and Enamelin. By integrating developmental signaling information from human enamel formation, the iPSC-derived ameloblast organoids successfully recapitulate early differentiation stages, demonstrating how single-cell insights can inform biomimetic regeneration, although their *in vivo* integration and functional maturation remain to be validated.

Recently, Hasan et al. (2025) reported a biomimetic approach to recapitulate the structural features of dental enamel *in vitro* by precisely emulating the critical signaling microenvironment of enamel development. In this study, a tunable supramolecular protein matrix was engineered using elastin-like recombinamers. Incorporation of calcium ions (Ca^2+^), combined with drying-induced molecular crowding, enabled the matrix to faithfully replicate the cross-β-sheet fibrillar architecture characteristic of natural amelogenin. This biomimetic matrix can be stably applied to the surface of damaged enamel, directing the epitaxial growth of highly ordered fluorapatite nanocrystals. As a result, the regenerated enamel exhibits remarkable fidelity to native tissue in both microstructural features—including prismatic and aprismatic enamel—and macroscopic mechanical properties, such as hardness, elastic modulus, and wear resistance.

Together, these findings provide a compelling proof-of-concept for human enamel regeneration and lay the groundwork for future translational applications. Despite advances in organoid and biomimetic strategies, the absence of a persistent enamel stem cell niche and the challenge of achieving seamless integration of newly formed enamel with pre-existing tooth structures, including native enamel and dentin, remain major hurdles for *in vivo* regeneration.

## 4 Cellular foundations underlying dentin-pulp and periodontal development and their regenerative approaches

### 4.1 Single-cell transcriptomic insights into early human dental mesenchyme heterogeneity

Alghadeer et al. (2023) characterized the heterogeneity of human dental mesenchymal cells during early tooth development (9–22 gestational weeks). Six distinct mesenchymal populations were identified: dental papilla (DP), pre-odontoblasts (POB), odontoblasts (OB), sub-odontoblasts (SOB), ectomesenchyme (DEM), and dental follicle (DF). Gene Ontology (GO) analysis revealed that DP and DEM are enriched for signaling, morphogenesis, developmental initiation, and patterning pathways, suggesting their roles as progenitor populations (Alghadeer et al., 2023). POB were characterized by proliferative and fate-determining signatures; DF cells displayed enrichment for genes involved in extracellular matrix formation; SOB expressed genes associated with cell aggregation, motility, and migration; while OB exhibited gene programs linked to odontogenesis, dental tissue formation, and mineralization (Alghadeer et al., 2023).

Pseudotime analysis further confirmed the presence of two progenitor populations within the dental mesenchyme: DP and DF. DP gives rise to POB and OB, whereas DF generates sparse DF-like cells (Alghadeer et al., 2023). Both progenitors are derived from PRRX1^+^ DEM cells (Alghadeer et al., 2023). At 13 gestational weeks, the dental pulp is primarily derived from DP, with DEM localized at the apical region of the pulp. Intriguingly, sparse DF-like cells are already present in the early dental pulp (prior to 13 weeks), suggesting a potential contribution of DF to pulp and dentin development. As tooth development progresses and progenitor density declines, the fate of OB lineage is largely established between 13 and 20 weeks (Alghadeer et al., 2023). By 19 weeks, the pulp contains a mixed population of sparse DF-like cells and POB, with OB positioned at the incisal edge; under injury, OB can be replenished by the underlying sparse DF-like cells, whereas during normal development, OB are predominantly derived from POB (Alghadeer et al., 2023).

In the study by Shi et al. (2024), the dental mesenchyme of human tooth germs from 17–24 gestational weeks was divided into seven subpopulations: SFRP1^+^/SOSTDC1^+^/SMOC2^+^ apical pulp; TNC^+^/DKK3^+^/HEY1^+^ DP; FRZB^+^/FGF3^+^/TWIST2^+^ DP; FGF3^+^/TWIST2^+^ OB and DKK3^+^/FBN2^+^ OB; IGFBP5^+^/SPON1^+^/FOXF1^+^ DF; and GDF10^+^/COL12A1^+^ DF. However, the interrelationships among these populations and their specific functional roles remain largely unexplored.

### 4.2 Single-cell profiling of postnatal human dental papilla

To date, single-cell RNA sequencing has identified eight major cell types within the postnatal human DP, including fibroblasts, odontoblasts, mesenchymal stem cells, monocytes/macrophages, lymphocytes, endothelial cells, glial cells, and proliferating cells (Ren et al., 2022). Notably, early mesenchymal stem cell populations exhibit expression of SEPTIN genes, which may endow these cells with enhanced proliferative and differentiation potential (Ren et al., 2022).

To further explore the osteogenic potential of DP-derived mesenchymal stem cells, Weng et al. (2025) employed scRNA-seq to investigate transcriptional changes during chemically induced osteogenesis, identifying a DIO2^+^ subpopulation with pronounced osteogenic activity. Moreover, overexpression of DIO2 enhanced the cranial bone regenerative capacity of DP mesenchymal stem cells. However, the isolation and *in vitro* culture system for DIO2^+^ cells have not yet been established, which may substantially limit the translational relevance of these findings. (summarized in Table 2). The identification of DP and DF progenitors with distinct transcriptional signatures suggests opportunities for selectively activating these populations to enhance dentin or periodontal regeneration.

**TABLE 2:** Cellular subpopulations of human dental mesenchyme and periodontal tissues and their characteristics.

Cell type	Tissue localization	Markers	Function	Lineage relationship	References
Pre-odontoblast	Peripheral dental pulp	Wnt10a^+^	Precursor cells for odontoblasts	Derived from DP; differentiates into OB during normal development	Alghadeer et al. (2023)
Odontoblast	Outermost layer of dental pulp (incisal edge)	Smpd3^+^	Functionally mature odontoblasts; responsible for dentin formation	Mainly derived from POB differentiation; can be generated from SOB upon injury	Alghadeer et al. (2023)
Sub-odontoblast	Layer beneath OB	No specific markers have been reported so far	Reserve cell population; can differentiate into OB upon injury	Derived from DF; present in early pulp, suggesting DF involvement in pulp/dentin development	Alghadeer et al. (2023)
Dental papilla Subtype 1 cell	Dental papilla	TNC^+^/DKK3^+^/HEY1^+^	Function unclear	Intercellular relationships and specific functions require further discussion	Shi et al. (2024)
Dental papilla Subtype 2 cell	Dental papilla	FRZB^+^/FGF3^+^/TWIST2^+^	Function unclear	Intercellular relationships and specific functions require further discussion	Shi et al. (2024)
Odontoblast Subtype 1 cell	Odontoblast layer	FGF3^+^/TWIST2^+^	Function unclear	Intercellular relationships and specific functions require further discussion	Shi et al. (2024)
Odontoblast Subtype 2 cell	Odontoblast layer	DKK3^+^/FBN2^+^	Function unclear	Intercellular relationships and specific functions require further discussion	Shi et al. (2024)
Dental follicle Subtype 1 cell	Dental follicle	IGFBP5^+^/SPON1^+^/FOXF1^+^	Function unclear	Intercellular relationships and specific functions require further discussion	Shi et al. (2024)
Dental follicle Subtype 2 cell	Dental follicle	GDF10^+^/COL12A1^+^	Function unclear	Intercellular relationships and specific functions require further discussion	Shi et al. (2024)
DIO2^+^ cell subpopulation	Apical papilla	DIO2^+^	Exhibits significant osteogenic activity; DIO2 transfection enhances calvarial regeneration capacity	Isolation and *in vitro* culture systems not established, limiting clinical significance	Weng et al. (2025)
Dental follicle stem cells	Dental follicle tissue	SPARC^+^/POSTN^+^	Primary effector cells for periodontal tissue formation (cementum, periodontal ligament, alveolar bone)	Tissue comprises endothelial cells, Schwann cells, immune cellsetc.	Liu J et al., 2025, Liu N et al., 2025
Dental progenitor cells	Dental follicle tissue	PDGFRA^+^	Differentiate into periodontal tissue components; regulated by endothelial PDGFBB signaling to promote angiogenesis	Culturing PDGFRA + cell aggregates promotes coupling of angiogenesis and osteogenesis in periodontal defects, accelerating periodontal bone regeneration	Liu J et al. (2025)

### 4.3 Postnatal human periodontal tissue development at the cellular level

Single-cell RNA sequencing of postnatal human dental follicle tissue has identified multiple cell types, including endothelial cells, Schwann cells, pericytes, T cells, B cells, plasma cells, macrophages, epithelial cells, and dental follicle stem cells (DFSCs) characterized by expression of SPARC and Periostin (POSTN) (Liu N et al., 2025; Liu J et al., 2025). SPARC^+^/POSTN^+^ DFSCs serve as the primary effector cells driving the formation of periodontal tissues, including cementum, the periodontal ligament, and alveolar bone (Liu N et al., 2025; Liu J et al., 2025). Furthermore, Liu N et al. (2025) identified PDGFRA as a marker for dental progenitor cell populations within DFSCs. In addition to differentiating and directly contributing to periodontal tissue formation, PDGFRA^+^ DFSCs are regulated by endothelial cell-derived PDGF-BB signaling, thereby promoting the formation of CD31^+^/EMCN^+^ vasculature (Liu N et al., 2025). Additionally, Liu J et al. (2025) cultured PDGFRA^+^ DFSCs aggregates to enhance the coupling of angiogenesis and osteogenesis in periodontal defects, thereby accelerating alveolar bone regeneration.

Another study established gene regulatory networks (GRNs) among dental follicle cells and identified four regulatory modules involved in tooth development and microenvironmental regulation, highlighting how vascular and immune components coordinate periodontal development via Collagen-CD44 and ANGPTL1-ITGA/ITGB signaling (Liu N et al., 2025). Although these studies (Liu J et al., 2025; Liu N et al., 2025) have significantly advanced our understanding of postnatal human periodontal development and proposed novel regenerative strategies, the effector cell populations responsible for the three major periodontal components—cementum, periodontal ligament, and alveolar bone—as well as the differentiation trajectories of DFSCs, remain largely unexplored.

### 4.4 Cellular architecture of mouse dental pulp and dentin during development

Over the past 2 decades, studies using genetic mouse models have identified several critical cell populations involved in incisor and molar development. Sensory nerves within the neurovascular bundle (NVB) secrete Shh protein, which activates Gli1^+^ perivascular cells and promotes the generation of all dental mesenchymal cells, playing an essential role in the development of mouse dental pulp and dentin (Zhao et al., 2014). Chen S et al. (2020) identified a Runx2^+^/Gli1^+^ subpopulation in adult mouse incisors that regulates the proliferation and differentiation of TACs and modulates incisor growth rate via IGF signaling, thereby contributing to the maintenance of the MSC niche.

PRX1 is a commonly used marker for mesenchymal stem cells or progenitor populations in craniofacial development (ten Berge et al., 2001). Zhao et al. (2024) using scRNA-seq, revealed that during the development of the first mouse M from embryonic day 13.5 to postnatal day 7.5, Prx1^+^ cells initially condense and display a specific distribution within the dental mesenchyme; however, their numbers decline by the bell stage. This finding provides preliminary evidence for a potential association between Prx1^+^ cells and tooth morphogenesis. Arai et al. (2017) proposed that Prx1^+^ cells regulate molar morphogenesis by secreting Wnt5a, while simultaneously promoting the proliferation of dental mesenchymal cells. However, throughout the observed stages of mouse tooth development, Prx1^+^ cells were not prominently detected in the third molar germ, suggesting that additional stem cell subpopulations may contribute to molar morphogenesis (Arai et al., 2017).

The development and widespread application of scRNA-seq has provided researchers with a more comprehensive understanding of the cellular foundations underlying mouse tooth development. Shan et al. (2025) revealed that mouse M formation is orchestrated by a spatially organized core of Cd24a^+^/Pax9^+^ progenitor cells, in which Cd24a^+^ cells give rise to the dental pulp-dentin complex, while Pax9^+^ cells primarily contribute to periodontal tissues. Guided by alveolar bone-derived platelet-derived growth factor subunit B (PDGFB), Cd24a^+^/Pax9^+^ cells progressively concentrate in the root region during the transition from crown to root, migrating collectively to form the tooth root. Notably, a similar population of Cd24a^+^/PAX9^+^ cells has also been observed during human tooth development.

Additionally, Jing et al. (2022) performed scRNA-seq on mouse M from embryonic day 13.5 to postnatal day 7.5. At E13.5, the dental mesenchyme expressed Tfap2b and Lhx6, representing a relatively homogeneous progenitor population at this stage (Jing et al., 2022). By E14.5, Pax9^+^ dental mesenchymal cells could be further subdivided into Crym^+^/Egr3^+^/Fgf3^+^ DP cells and DF cells (Jing et al., 2022). At E16.5, when the molars reach the bell stage, cellular heterogeneity emerges within both the DP and DF compartments: the DP population segregates into Lmo1^+^/Fgf3^+^/Smpd3^+^ cells in the coronal domain and Lhx6^+^/Fst^+^/Gldn^+^ cells in the apical domain, while the DF similarly evolves into two distinct cellular domains.

**FIGURE 3:**
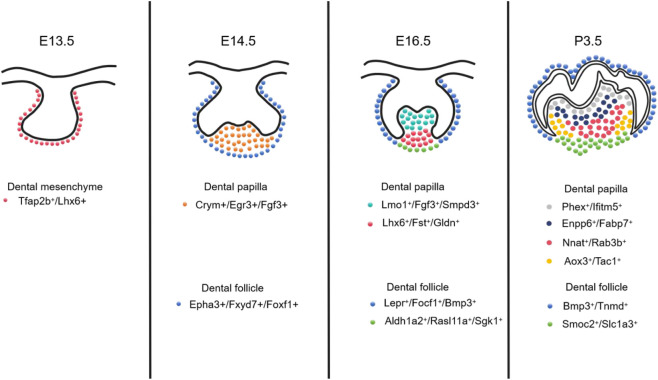
At the E13.5 stage, the dental mesenchyme is primarily composed of relatively homogeneous progenitor cells expressing Tfap2b^+^/Lhx6^+^. By E14.5, the cells begin to undergo initial differentiation: dental papilla cells express Crym^+^/Egr3^+^/Fgf3^+^, while dental follicle cells express Epha3^+^/Fxyd7^+^/Foxf1^+^. During E16.5 (the bell stage), cellular heterogeneity increases significantly: the dental papilla differentiates into coronal domain Lmo1^+^/Fgf3^+^/Smpd3^+^ cells and apical domain Lhx6^+^/Fst^+^/Gldn^+^ cells, while the dental follicle similarly divides into two distinct cellular domains.By postnatal day 3.5 (P3.5), as tooth root formation begins, six distinct cellular subpopulations become apparent within the dental mesenchyme. The dental papilla further differentiates into: Phex^+^/Ifitm5^+^ osteoblasts, Enpp6^+^/Fabp7^+^ coronal dental papilla cells, Nnat^+^/Rab3b^+^ middle dental papilla cells, and Aox3^+^/Tac1^+^ apical dental papilla cells. The dental follicle consists of two distinct subpopulations. This spatial and molecular architecture remains largely stable by P7.5, indicating that the cellular framework for root formation is already established by P3.5.

At postnatal day 3.5, mouse M roots are poised to initiate formation, and six distinct cell populations can be observed within the dental mesenchyme, including two DF subpopulations (Jing et al., 2022). The DP further segregates into Phex^+^/Ifitm5^+^ OB, Enpp6^+^/Fabp7^+^ coronal DP, Nnat^+^/Rab3b^+^ middle DP, and Aox3^+^/Tac1^+^ apical DP. By postnatal day 7.5, the spatial organization and corresponding marker genes within the dental mesenchyme remain largely consistent with those observed at P3.5, indicating that the cellular architecture for root formation is already established at P3.5 (Jing et al., 2022). These findings provide a critical foundation for understanding cellular mechanisms underlying mouse M development and offer valuable insights for advancing tooth regenerative strategies (Figure 3).

### 4.5 Mouse periodontal tissue development at single-cell resolution

Gli1^+^ progenitor cells are directly responsible for mouse periodontium development (Pei et al., 2024), and those residing in the periodontal ligament (PDL) can further contribute to alveolar bone regeneration (Shalehin et al., 2022). Axin2^+^ cells play a pivotal role in rapid cementum formation during periodontal development (Xie et al., 2019). In addition, Prx1^+^ cells are implicated in periodontium formation; during periodontal reconstruction in transplanted mouse teeth, Prx1^+^ cells originating from the recipient alveolar bone act as perivascular cells to support angiogenesis (Gong et al., 2022).

Based on scRNA-seq, Epha3^+^/Fxyd7^+^/Foxf1^+^ DF cells were first detected in mouse M at embryonic day 14.5 (Jing et al., 2022). At embryonic day 16.5, when mouse M reach the bell stage, DF cells exhibit pronounced heterogeneity, segregating into Lepr^+^/Foxf1^+^/Bmp3^+^ lateral-domain DF and Aldh1a2^+^/Rasl11a^+^/Sgk1^+^ apical-domain DF (Jing et al., 2022). At postnatal day 3.5, as mouse M roots are poised to initiate formation, dental follicle (DF) cells remain segregated into two domains: Bmp3^+^/Tnmd^+^ lateral-domain DF and Smoc2^+^/Slc1a3^+^ apical-domain DF. The spatial organization of DF closely mirrors that observed at E16.5, indicating that the cellular architecture of the DF is already established by the bell stage (Jing et al., 2022). Lineage-tracing experiments demonstrate that Lepr^+^ DF cells give rise to periodontal tissues, including the PDL and alveolar bone (Jing et al., 2022). These findings indicate that Lepr^+^ and Gli1^+^ cells likely serve as progenitor populations during mouse periodontium development.

The distribution of PTHrP^+^ cells within the dental follicle (DF) is critical for mouse root formation and tooth eruption (Ono et al., 2016). Takahashi et al. (2019) used scRNA-seq to analyze the heterogeneity of PTHrP^+^ DF cells and found that during root development, PTHrP^+^ cells differentiate on decellularized cementum into osteoblasts, periodontal ligament cells, and alveolar crypt osteoblasts. Consequently, PTHrP^+^ cells are considered progenitor populations within the DF. Loss of the PTHrP receptor (PPR) causes these progenitors to aberrantly differentiate into cementum-like cells, leading to premature formation of cellular cementum on the root surface, accompanied by upregulation of Mef2c and extracellular matrix proteins, ultimately resulting in disruption of normal periodontal attachment and failed tooth eruption (Liu N et al., 2025). In the study by Nagata et al. (2021) three lineage populations within the dental follicle (DF) were further characterized: Tubb3^+^/PTHrP^+^ cementoblasts, Phex^+^/Ifitm5^+^ osteoblasts, and Scx^+^/Postn^+^ PDL cells. During periodontium development, the Scx^+^ PDL cell population may differentiate into osteoblasts and fibroblasts, suggesting that Scx serves as a marker for mouse periodontal ligament stem cells (PDLSCs) (Liu J et al., 2025). In contrast, Tubb3^+^/PTHrP^+^ cementoblast-lineage cells appear to remain lineage-restricted, indicating that cementoblasts possess distinct characteristics from osteoblasts (Liu N et al., 2025).

Currently, three major progenitor populations within the DF have been identified: Gli1^+^, Lepr^+^, and PTHrP^+^ DF progenitors, yet the relationships among these populations remain poorly understood. Notably, Nagata et al. (Nagata et al., 2025) reported that PTHrP^+^ DF cells represent a subpopulation of Gli1^+^ cells. Although the mechanisms by which Gli1^+^ cells give rise to PTHrP^+^ cells remain unclear, the fate regulation of PTHrP^+^ DF cells has been elucidated (Nagata et al., 2025). During early mouse periodontium development, the HERS activates Hedgehog-Smo-Foxf signaling within PTHrP^+^ DF cells, maintaining them in an undifferentiated, self-renewing state (Nagata et al., 2025). As development progresses and HERS gradually regresses, Hedgehog-Smo-Foxf signaling in PTHrP^+^ DF cells is attenuated, allowing their differentiation into osteoblast-lineage cells, ultimately contributing to alveolar bone formation (Nagata et al., 2025).

Notably, parathyroid hormone (PTH) secreted by the parathyroid gland and PTHrP within the DF play pivotal roles in mediating tooth eruption (Nagata et al., 2020; Gensure et al., 2005). A previous study demonstrated that PTHrP can increase the RANKL/OPG ratio in DF cells, thereby promoting osteoclastogenesis (Zhang et al., 2019). Additionally, secreted PTHrP can act in both paracrine and autocrine manners to enhance the osteogenic differentiation of DFSCs (Pieles et al., 2022; Pieles et al., 2020). Collectively, these findings suggest that PTHrP^+^ DF cells not only contribute directly to periodontal tissue formation (Nagata et al., 2025) but may also facilitate tooth eruption and the differentiation of neighboring PTHrP-DF cells via PTHrP signaling (Nagata et al., 2020; Gensure et al., 2005; Zhang et al., 2019; Pieles et al., 2022; Pieles et al., 2020).

Through transgenic animal models and scRNA-seq, researchers have gained a relatively comprehensive and in-depth understanding of the cellular basis of mouse periodontium development. However, the periodontal regenerative potential of the three major DF progenitor populations—Gli1^+^, Lepr^+^, and PTHrP^+^ DF cells—remains unexplored. Moreover, whether these progenitor populations exist and function within the human dental follicle has yet to be validated.

### 4.6 Developmental insights and signal-guided approaches for dentin regeneration

Recent single-cell and spatial transcriptomic analyses have provided unprecedented insight into human dentinogenesis, revealing the precise spatiotemporal coordination of epithelial–mesenchymal interactions (Wei et al., 2026). DE cells secrete WNT and NOTCH ligands in a dynamic, stage-specific manner to regulate the differentiation of DLX6-AS1^+^ DP progenitors into odontoblasts (Wei et al., 2026). Temporal analysis indicates a relay-like mechanism, with early-stage WNT signaling promoting progenitor specification and later-stage NOTCH activation driving odontoblast maturation and dentin matrix deposition. Spatial mapping confirmed that DLX6-AS1^+^ DP cells are positioned in close proximity to DE cells, enabling targeted reception of inductive signals. Building on these developmental insights, sequential WNT-NOTCH stimulation of DLX6-AS1^+^ dental mesenchymal stem cells (DMSCs) and DPSCs significantly enhanced odontogenic differentiation *in vitro*, as demonstrated by elevated DSPP expression, increased alkaline phosphatase activity, and robust mineralization (Wei et al., 2026). Moreover, tissue recombination and *in vivo* transplantation assays showed that this sequential stimulation enables these progenitors to form well-organized, tubular dentin structures, closely resembling native dentin architecture. These findings establish a developmentally informed, signal-guided strategy for dentin regeneration, highlighting the translational potential of DLX6-AS1^+^ mesenchymal populations as seed cells for clinical applications (Wei et al., 2026). Nonetheless, current strategies remain limited by incomplete replication of the dentin microenvironment, including vascularization, innervation, and epithelial–mesenchymal interactions, suggesting that future work should integrate these components to achieve fully functional dentin regeneration.

### 4.7 Regeneration of the mouse pulp-dentin complex via Cd24a^+^ cell spheroids

As previously discussed, mouse Cd24a^+^ cells are capable of differentiating into the pulp–dentin complex, and Cd24a^+^ cells have also been observed in the human dental papilla (Shan et al., 2025). Importantly, the pulp regenerative potential of mouse Cd24a^+^ cells has been previously confirmed (Shan et al., 2025; Chen H et al., 2020). Chen S et al. (2020) identified and isolated a distinct population of Cd24a^+^ multipotent dental pulp regenerative stem cells (MDPSCs) from mouse dental papilla using three-dimensional spheroid culture. These Cd24a^+^ MDPSCs exhibit enhanced osteogenic and dentinogenic differentiation capacity, marked by SP7 expression, and are capable of forming dentin-like structures and neurovascular networks *in vivo*, closely recapitulating the morphology of native mouse teeth.

## 5 Single-cell transcriptomics reveals key mesenchymal subpopulations in mouse tooth regeneration

During embryonic days 11–13 in mice, odontogenic signals are transferred from the dental epithelium to the underlying mesenchyme. Previous studies have demonstrated that non-dental epithelia—including human keratinocytes (Ruan et al., 2017), human gingival epithelial cells (Angelova Volponi et al., 2013), and even epithelial sheets derived from human embryonic stem cells (Cai et al., 2013) —can respond to odontogenic mesenchymal cues, collectively contributing to tooth formation. Wang et al. (2022) reported that at embryonic day 12.5 in mice, cellular differences between incisor and molar tooth germs become readily distinguishable, with pronounced heterogeneity observed primarily in mesenchymal populations rather than epithelial cells. At this stage, Hand1, Alx1/3, and Pax3 act as incisor-specific regulatory genes driving mesenchymal development, whereas Tbx15, Lhx6, and Tfap2b serve as molar-specific mesenchymal regulators (Wang et al., 2022). These findings suggest that tooth-type specification in mice may largely be dictated by odontogenic mesenchyme endowed with developmental signaling cues. Furthermore, during the bell stage of mouse tooth development, Wang et al. (2022) observed that CD24^+^ and Plac8^+^ mesenchymal cells are spatially segregated within the dental papilla, with CD24^+^ cells predominantly occupying the upper layer and Plac8^+^ cells localizing to the pre-odontoblast layer. Both populations are capable of independently inducing non-dental epithelia to form tooth-like structures, although Plac8^+^ cells exhibit a markedly higher odontogenic potential.

Hu et al. (2022) using scRNA-seq, identified the emergence of the mouse dental papilla at embryonic day 12.5, specifically marked by Msx1 and Sdc1. Within the dental niche, they further discovered a previously unrecognized subpopulation of Msx1^+^/Sox9^+^ microenvironmental cells (Hu et al., 2022). Notably, these Msx1^+^/Sox9^+^ cells exhibit a markedly higher odontogenic potential than the Msx1^+^/Sdc1^+^ dental papilla cells, forming clustered tooth-like structures and potentially representing the progenitors of dental papilla cells (Hu et al., 2022).

These findings indicate that differential odontogenic potential among mesenchymal subpopulations is already established in early tooth organogenesis. More importantly, they provide critical insights into the fundamental cellular constituents for dental tissue engineering and regenerative strategies.

## 6 Conclusions and perspectives

Tooth development is an intricately orchestrated process, and scRNA-seq has proven invaluable for comprehensively dissecting the cellular landscape, lineage trajectories, fate maintenance mechanisms, and inter-subpopulation signaling within epithelial–mesenchymal compartments. These deeper insights into tooth organogenesis provide a foundational framework for odontogenic iPSC induction, organoid formation, and the isolation and *in vitro* expansion of key developmental cell populations, thereby offering a promising avenue toward partial or complete functional tooth regeneration.

To date, researchers have applied single-cell RNA sequencing to mouse tooth germs across various developmental stages, as well as to postnatal dental pulp and periodontal ligament (Lee et al., 2022), generating comprehensive single-cell atlases for these distinct tissues. Although there are certain similarities in the developmental patterns and sequences of mouse M and human teeth, differences in tooth size, morphology, and recently discovered cellular and molecular mechanisms (Hermans et al., 2022) continue to drive efforts to gain insights into human dental development. This knowledge is crucial for advancing strategies for human tooth regeneration. Researchers have successfully performed scRNA-seq on early-stage human embryonic tooth buds (Alghadeer et al., 2023; Shi et al., 2024), providing valuable cellular data on the early stages of human tooth development. However, due to ethical constraints, data on tooth buds from later-stage embryos remain inaccessible. Additionally, the lack of methods for tracking and validating key cell populations during human tooth development significantly hinders further exploration and understanding of this process.

Research on the postnatal development of human third molars has largely mitigated the challenge of obtaining tooth bud data from late-stage embryos. While scRNA-seq studies of the postnatal human dental papilla and dental follicle have provided substantial insights into the cellular heterogeneity and key signaling pathways (Ren et al., 2022; Weng et al., 2025; Liu J et al., 2025; Liu N et al., 2025; Shi et al., 2021), the full heterogeneity of the stem cell populations within these tissues remains unexplored. Furthermore, the relationships between these cells and the progenitor populations identified in early-stage tooth buds have yet to be investigated. Given that third molars and premolars, often extracted due to factors such as orthodontics or impacted wisdom teeth, contain a wealth of mesenchymal stem cells, they offer a reliable source of cells for regenerative medicine. Therefore, continued research on postnatal human tooth development holds immense potential for advancing our understanding of tooth development and regeneration.

Beyond defining cellular hierarchies, current studies underscore the therapeutic potential of distinct dental progenitor subsets for regenerative applications, including partial or full tooth reconstruction. These findings highlight that successful translation relies not only on isolating and expanding progenitors, but also on precisely recapitulating the spatiotemporal signaling and microenvironmental cues that guide odontogenesis. Despite promising proof-of-concept studies, several major translational barriers remain. First, the inability of current *in vitro* and organoid systems to fully mimic the dynamic multicellular niche of developing teeth—including vascularization, innervation, immune interactions, and extracellular matrix remodeling—limits both functional maturation of progenitor-derived tissues and faithful recapitulation of *in vivo* morphogenesis. Second, the lack of robust lineage-tracing and functional validation tools in human tissues constrains definitive characterization of progenitor identity, differentiation potential, and long-term safety, making extrapolation from animal models necessary but inherently uncertain. Third, heterogeneity in progenitor populations and differentiation outcomes presents critical challenges for reproducibility, scalability, and standardization of regenerative constructs, complicating translation toward clinically viable therapies. Collectively, these barriers underscore key future directions: development of biomimetic and vascularized organoid systems, establishment of human-relevant lineage and functional assays, and strategies to standardize progenitor expansion and differentiation. Importantly, although these studies provide foundational insights, there remains a paucity of literature systematically leveraging identified dental progenitor subsets for functional tooth regeneration, indicating that translational and mechanistic exploration of these populations is still in its infancy. Addressing these challenges will require integrated approaches combining multimodal single-cell multi-omics, advanced biomaterials that recapitulate developmental niches, and computational or AI-guided frameworks to optimize differentiation and regenerative outcomes.

In addition to scRNA-seq, other technologies such as single-cell proteomics (Mund et al., 2022), single-cell metabolomics (Lanekoff et al., 2022), and even artificial intelligence (AI) (Carrillo-Perez et al., 2022) have opened new avenues for more comprehensive analyses of human tooth development. These advancements provide fresh hope for achieving full tooth regeneration.
